# Antioxidant Fusion Protein SOD1-Tat Increases the Engraftment Efficiency of Total Bone Marrow Cells in Irradiated Mice

**DOI:** 10.3390/molecules26113395

**Published:** 2021-06-03

**Authors:** Ting Bei, Xusong Cao, Yun Liu, Jinmei Li, Haihua Luo, Lin Huang, Tian Tian, Lei Li, Yong Jiang

**Affiliations:** Guangdong Provincial Key Laboratory of Proteomics and State Key Laboratory of Organ Failure Research, School of Basic Medical Sciences, Southern Medical University, Guangzhou 510515, China; bettine-200390@foxmail.com (T.B.); a670555024@126.com (X.C.); sysliuyun@126.com (Y.L.); jmli12@163.com (J.L.); btx_lhh@126.com (H.L.); huanglin202104@163.com (L.H.); d5tian218@163.com (T.T.); lilei747667911@163.com (L.L.)

**Keywords:** cells penetrating peptides, bone marrow transplantation, reactive oxygen species, engraftment, superoxide dismutase

## Abstract

Total body irradiation is a standard procedure of bone marrow transplantation (BMT) which causes a rapid increase in reactive oxygen species (ROS) in the bone marrow microenvironment during BMT. The increase in ROS reduces the engraftment ability of donor cells, thereby affecting the bone marrow recovery of recipients after BMT. In the early weeks following transplantation, recipients are at high risk of severe infection due to weakened hematopoiesis. Thus, it is imperative to improve engraftment capacity and accelerate bone marrow recovery in BMT recipients. In this study, we constructed recombinant copper/zinc superoxide dismutase 1 (SOD1) fused with the cell-penetrating peptide (CPP), the trans-activator of transcription (Tat), and showed that this fusion protein has penetrating ability and antioxidant activity in both RAW264.7 cells and bone marrow cells in vitro. Furthermore, irradiated mice transplanted with SOD1-Tat-treated total bone marrow donor cells showed an increase in total bone marrow engraftment capacity two weeks after transplantation. This study explored an innovative method for enhancing engraftment efficiency and highlights the potential of CPP-SOD1 in ROS manipulation during BMT.

## 1. Introduction

Total body irradiation during bone marrow transplantation (BMT) causes a rapid increase in reactive oxygen species (ROS) in bone marrow niches. Increased ROS leads to reduced engraftment capability of the transplanted cells [1], thereby affecting bone marrow recovery after BMT. In the early weeks following transplantation, the recipient is at high risk for severe infection due to bone marrow suppression [2,3]. Thus, there is a need to improve engraftment efficiency and to accelerate bone marrow recovery in recipients as early as possible after BMT. Intravenous administration of the antioxidant N-acetyl-L-cysteine (NAC) or ROS-detoxifying enzymes or overexpression of ROS-detoxifying enzymes in donor hematopoietic stem cells (HSCs) has been reported to enhance the engraftment capacity of transplanted HSCs in mice. However, the evidence is very limited [4,5]. In addition, overexpression strategies have potential risks due to the genomic fusion of exogenous antioxidant genes, which may lead to the long-term dysfunction of HSCs. Thus, novel methods need to be developed for ROS manipulation to improve the outcome of BMT and accelerate bone marrow and immune system reconstitution after transplantation.

Cell penetrating peptides (CPPs) have been studied for over three decades [6]. CPPs are an effective intracellular transport tool for bioactive molecules and they show promising applications in cell biology, immunology, drug development, gene biotherapy, and targeted tumor therapy [6]. Compared with overexpression strategies that require genetic modification, CPPs are safer than integrating exogenous genes into the genome of cells [5]. The sequence of CPP was first found in the transactivator of transcription (Tat) protein of human immunodeficiency virus (HIV) [7]. The protein transduction domain (PTD) refers to the sequence with penetrating capacity. For example, the basic domain YGRKKRRQRRR of Tat (49–57) can rapidly translocate through the plasma membrane [8]. Recombinant fusion proteins combined with PTD sequences or artificial CPPs such as arginine-rich peptides have the capability to penetrate cells. Previous studies have investigated the potential for the application of CPPs in various disease models, including cancer, stroke, psoriasis, etc. [9,10].

Although numerous studies have used CPPs to transport molecules into cells in disease models, intervention strategies with CPPs to improve engraftment following BMT have not been thoroughly investigated, but may have great potential in this context. Lewin et al. used Tat peptides combined with superparamagnetic nanoparticles to track hematopoietic cells without affecting cell viability and differentiation ability [11]. Song et al. reported that the administration of rFOXN1, a member of the winged helix/forkhead box transcription factor family, fused with a CPP into congenic or allogeneic HSC transplantation recipient mice resulted in enhanced thymopoiesis and an increase in peripheral T cells [12]. However, there are no reports on the application of CPPs to BMT by directly treating total bone marrow (TBM) donor cells with CPP-fused recombinant antioxidant protein.

Antioxidant enzymes, including copper/zinc superoxide dismutase 1 (SOD1), glutathione peroxidase, and catalase, are the major cellular enzymes for ROS metabolism. In particular, SOD1, a homodimeric metalloenzyme, is critical for protecting cells from damage caused by ROS in a wide range of eukaryotic species. Each subunit of SOD1 has a Cu/Zn site, which catalyzes the conversion of superoxide (O_2_^−^) to molecular oxygen (O2) and hydrogen peroxide (H_2_O_2_) [13,14]. Overexpression of ROS-detoxifying enzymes in donor cells has been reported to increase engraftment after BMT by reducing ROS in the bone marrow niche [4,5]. However, the utility of integrating the cell penetration technique and antioxidant enzymes needs further investigation. In the present study, we developed an innovative method to increase engraftment efficiency by treating donor cells with the CPP fusion protein SOD1-Tat. With a traditional strategy of total bone marrow transplantation (tBMT) in lethally irradiated mice [15], we demonstrated that the CPP delivery method has great potential in ROS manipulation during bone marrow transplantation.

## 2. Results

### 2.1. SOD1-Tat Shows Penetrating Ability in RAW264.7 Cells and Bone Marrow Cells

**Figure 1:**
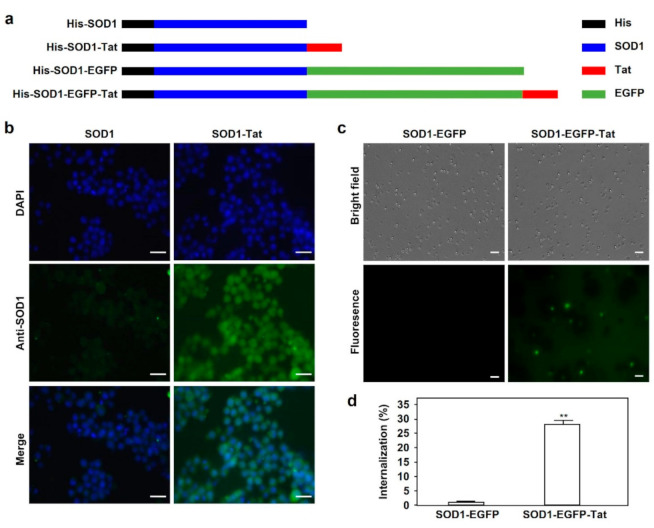
Tat-mediated cell-penetrating activity of SOD1 fusion proteins. (**a**). Schematic structures of His-SOD1, His-SOD1-Tat, His-SOD1-EGFP, and His-SOD1-EGFP-Tat fusion proteins expressed in the Rosetta 2(DE_3_) strain of E. coli. (**b**). Tat-mediated cell penetration of SOD1 fusion proteins in RAW264.7 cells. RAW264.7 cells were incubated with 5 μΜ SOD1 or SOD1-Tat fusion protein at 37 °C for 1 h. After incubation with a specific anti-SOD1 antibody and the corresponding secondary antibody, the cells were observed for protein internalization by fluorescence microscopy. Bars represent the length of 40 μΜ. (**c**). Tat-mediated cell penetration of SOD1 fusion proteins in bone marrow cells. Bone marrow cells were isolated from mouse femurs and incubated with 5 μΜ SOD1-EGFP or SOD1-EGFP-Tat fusion protein at 37 °C for 1 h. Tat-mediated protein internalization was examined by fluorescence microscopy. Bars represent the length of 20 μΜ. (**d**). Quantitative analysis of Tat fusion protein internalization in bone marrow cells. The percentage of EGFP-positive cells in Figure 1c was calculated under a fluorescence microscope. Data are shown as mean ± SD and represent three independent experiments (*n* = 3). **: *p* < 0.01, versus SOD1-EGFP group.

To clarify whether the SOD1-Tat recombinant fusion protein can penetrate cells, we constructed prokaryotic plasmids with pET14b as a vector for the expression of His-tagged fusion proteins, including SOD1, SOD1-Tat, SOD1-enhanced green fluorescent protein (EGFP), and SOD1-EGFP-Tat (Figure 1a). The fusion proteins were purified from Rosetta 2(DE3) bacterial cells, and protein purity was confirmed by SDS-PAGE. After incubation of the fusion proteins for 1 h, the primary antibody against mouse SOD1 was used to detect internalized SOD1. As shown in Figure 1b, the internalization of SOD1-Tat into RAW264.7 cells was confirmed by fluorescence microscopy, and a noticeable difference in fluorescence intensity between SOD1 and SOD1-Tat-treated cells was observed.

To determine whether SOD1-Tat can be internalized into TBM cells, we isolated TBM cells from the tibias and femurs of mice. TBM cells were treated with 5 μΜ SOD1-EGFP-Tat or SOD1-EGFP recombinant proteins for 1 hr. We found that SOD1-EGFP-Tat was internalized into TBM cells at a rate of 30%, which is much higher than that of SOD1-EGFP (*p* < 0.01) (Figure 1c,d).

### 2.2. SOD1-Tat Reduces ROS in RAW264.7 Cells and Lineage-c-Kit^+^ (LK) Cells

**Figure 2:**
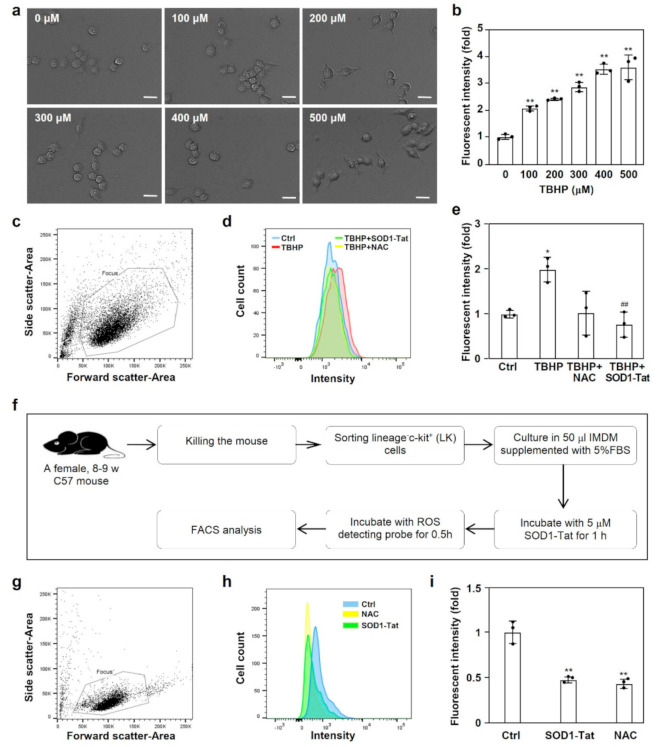
The antioxidant activity of the SOD1-Tat fusion protein. (**a**). Morphology of RAW264.7 cells challenged with tert-butyl hydroperoxide (TBHP) at different concentrations. Bars represent the length of 20 μΜ. (**b**). TBHP treatment increased the ROS levels in RAW264.7 cells. RAW264.7 cells were challenged with different concentrations of TBHP. The ROS level was measured by flow cytometry with a DCFHDA probe of the CellROX^®^ green assay kit. *: *p* < 0.05; **: *p* < 0.01, versus the control group. (**c**–**e**). SOD1-Tat pretreatment restrained ROS production in RAW264.7 cells challenged with TBHP. RAW264.7 cells were pretreated with 5 μΜ SOD1-Tat, 1 mM N-acetyl-L-cysteine (NAC), or PBS for 0.5 h. Then, 100 μΜ TBHP was added for another 0.5 h. The ROS level was measured by flow cytometry with a DCFHDA probe. The RAW264.7 cell population was selected by gating physical characteristics (**c**). Histogram of the fluorescence intensity of ROS in RAW264.7 cells (**d**). Blue line, control group; red line, TBHP group; green line, TBHP+SOD1-Tat group; yellow line, TBHP+NAC group. ROS production in RAW264.7 cells was quantitated according to the ratio of fluorescence intensity in the experimental versus control groups (**e**). Results are presented as mean ± SD and represent three independent experiments (*n* = 3). *, *p* < 0.05, versus control group; ##, *p* < 0.01, versus TBHP group. (**f**). Flow chart for the detection of ROS. LK cells were sorted from isolated TBM cells and cultured in 50 μL IMDM containing 5% FBS. After incubation with 5 μΜ SOD1-Tat, 1 mM NAC, or PBS for 1 h, the ROS level was measured by flow cytometry with a DCFHDA probe. (**g**–**i**). SOD1-Tat treatment decreased ROS production in LK cells. The live cell population was selected by gating physical characteristics (**g**). Histogram of the fluorescence intensity of ROS in LK cells (**h**). Blue line, control group; green line, SOD1-Tat group; yellow line, NAC group. ROS production in LK cells was quantitated according to the ratio of fluorescence intensity in the experimental versus control groups (**i**). Results are presented as mean ± SD, and represent three independent experiments (*n* = 3). **, *p* < 0.01, versus control group.

To determine whether the SOD1-Tat fusion protein has antioxidant activity, we pre-incubated RAW264.7 cells with the SOD1-Tat protein or PBS as a control. We used tert-butyl hydroperoxide (TBHP) as a ROS-inducing reagent. The morphology of RAW264.7 cells challenged with tert-butyl hydroperoxide (TBHP) at different concentrations is shown in Figure 2a. We found that TBHP dose-dependently increased cellular ROS in RAW264.7 cells (*p* < 0.01) (Figure 2b).

To test the effect of SOD1-Tat on the production of ROS in RAW264.7 cells, we performed flow cytometry to detect ROS in RAW264.7 cells after stimulation with TBHP for 30 min (Figure 2c,d). Intriguingly, compared with the control, SOD1-Tat dramatically reduced the level of ROS in RAW264.7 cells (*p* < 0.01) (Figure 2e).

To clarify whether the SOD1-Tat fusion protein reduces ROS in LK cells, hematopoietic precursor LK cells were sorted from TBM cells (Figure 2f,g). The flow cytometry results showed that, compared with the control, SOD1-Tat significantly reduced ROS in LK cells (*p* < 0.01) (Figure 2h,i).

### 2.3. SOD1-Tat Increases the Engraftment Ability of Total Bone Marrow Cells in Irradiated Mice

**Figure 3:**
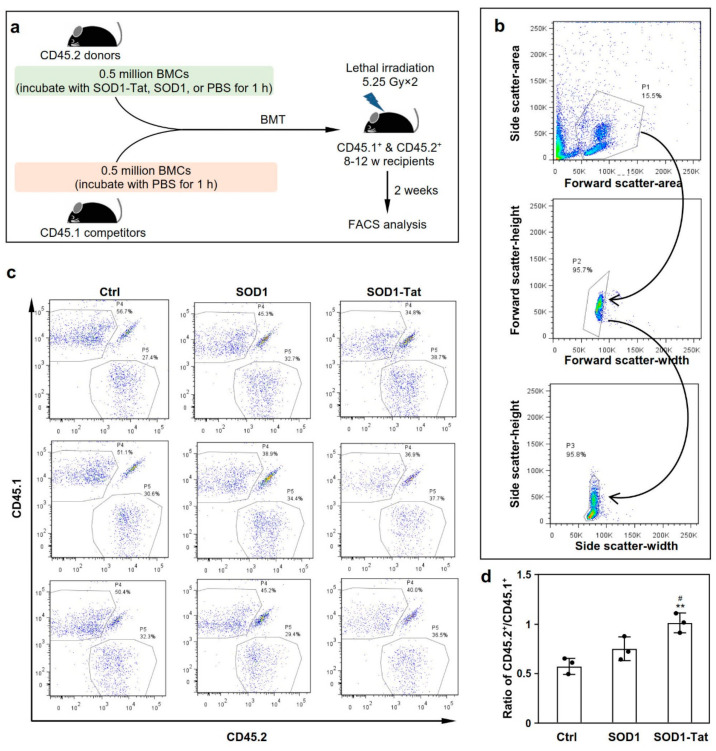
SOD1-Tat increases the total bone marrow engraftment capacity. (**a**). The experimental flow chart of competitive transplantation. After treatment with recombinant SOD1-Tat, SOD1, or PBS as a control, half a million CD45.2 BMCs were used to compete against an equal number of CD45.1 BMCs to reconstitute the hematopoietic compartment of a lethally irradiated recipient mouse (CD45.1^+^ & CD45.2^+^). Two weeks after transplantation, peripheral blood samples were collected retro-orbitally from the transplanted recipient mice for FACS analysis. (**b**). Living cell populations selected by gating on the physical characteristics of peripheral white blood cells. After two steps of selection according to cellular characteristics, population 3 was finally selected for FACS analysis. Cell clusters, cell debris, and other non-cell particles were excluded from the subsequent hematopoietic chimerism analysis. (**c**). FACS plots showing the percentage of CD45.1^+^ (P4) and CD45.2^+^ (P5) donor-derived WBCs in the peripheral blood two weeks after transplantation. The results represent three independent biological repeats (*n* = 3). (**d**). Ratio of CD45.2^+^ (P4) to CD45.1^+^ (P5) donor-derived WBCs. Results were presented as mean ± SD, *n* = 3. #: *p* < 0.05; **: *p* < 0.01, versus the control group.

We performed competitive transplantation to determine the effect of SOD1-Tat on the recovery of bone marrow and the immune system after BMT. After incubation with the fusion protein SOD1-Tat, SOD1, or PBS, 0.5 million BMCs from CD45.2^+^ mice were used for competition against an equal number of BMCs from a congenic CD45.1^+^ mouse to reconstitute the hematopoietic microenvironment of the recipient subjected to irradiation (Figure 3a). Two weeks after transplantation, we collected peripheral blood samples from the transplanted recipients for flow cytometry (Figure 3b). We found that SOD1-Tat-treated BMCs showed a better engraftment capacity than the control BMCs (*p* = 0.002), as indicated by the ratio of CD45.2/CD45.1 (Figure 3c,d). As expected, the SOD1-Tat group also showed a significant improvement in engraftment capacity in comparison with the SOD1 group (*p* = 0.019), while no significant difference was observed in engraftment capacity between the SOD1 and control groups (Figure 3c,d). These results indicate that SOD1-Tat significantly improves the engraftment capacity of BMCs early after transplantation.

### 2.4. SOD1-Tat Has No Significant Effect on Myeloid Cell Differentiation in Mice With BMT

**Figure 4:**
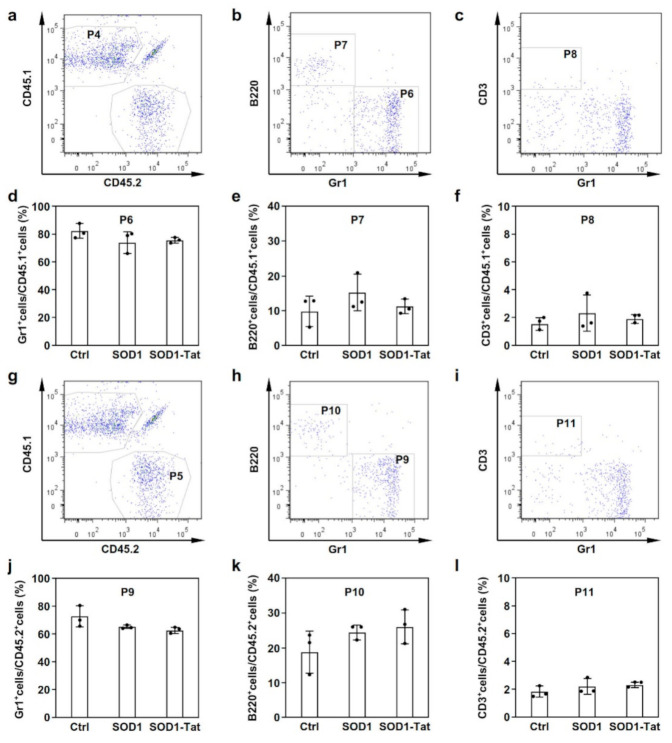
SOD1-Tat had no significant effect on myeloid cell differentiation in the recipient mice two weeks after BMT. (**a**). FACS plots showing chimerism in the indicated blood lineages at two weeks after transplantation. Population 4 (P4) represents CD45.1^+^ donor-derived WBCs. (**b**,**c**). Subpopulations of CD45.1^+^ cells gated for granulocytes (Gr1^+^), B lymphocytes (B220^+^), and T lymphocytes (CD3^+^). CD45.1^+^ cells (P4) were further divided into three different subpopulations, i.e., populations 6, 7 (**b**), and 8 (**c**), based on gating with cell markers for granulocytes (Gr1^+^), B lymphocytes (B220^+^), and T lymphocytes (CD3^+^). (**d**–**f**). The percentage ratios of Gr1^+^ (**d**), B220^+^ (**e**), or CD3^+^ (**f**) cells to CD45.1^+^ cells (P4). (**g**). Population 5 (P5) represents CD45.2^+^ donor-derived WBCs. (**h**,**i**). Subpopulations of CD45.2^+^ cells gated for granulocytes (Gr1^+^), B lymphocytes (B220^+^), and T lymphocytes (CD3^+^). CD45.2^+^ cells (P5) were further divided into three different subpopulations, i.e., populations 9, 10 (**h**), and 11 (**i**), based on gating with cell markers for granulocytes (Gr1^+^), B lymphocytes (B220^+^), and T lymphocytes (CD3^+^). (**j**–**l**). The percentage ratios of Gr1^+^ (**j**), B220^+^ (**k**), or CD3^+^ (**l**) cells to CD45.2^+^ cells (P5).

In addition, the chimerism of donor-derived CD45.1 or CD45.2 white blood cells (WBCs) was analyzed to determine the effect of SOD1-Tat on myeloid differentiation in mice with BMT. Three populations of WBCs, myeloid cells (Gr1^+^), B cells (B220^+^), and T cells (CD3^+^), were analyzed. We found that there were no significant differences in myeloid differentiation between the SOD1-Tat group and the control group in either the CD45.1 or the CD45.2 donor-derived WBCs (Figure 4). These results suggest that SOD1-Tat does not significantly affect myeloid differentiation in mice with BMT.

## 3. Discussion

In this paper, we reported that the SOD1-Tat fusion protein exhibited cell-penetrating ability and antioxidant activity in both RAW264.7 cells and bone marrow cells. Furthermore, TBM donor cells pretreated with SOD1-Tat showed an increased engraftment capability in irradiated recipient mice with BMT.

CPPs refer to peptides that exhibit cell-penetrating ability and have been reported to be promising in many scenarios, such as cell biology, immunology, and targeted therapy for cancers. The uptake mechanisms of CPPs are still under debate, but the two major mechanisms being considered are endocytosis and direct penetration [16]. As reported by Tunnemann et al., Tat was hypothesized to be prone to exert direct penetration when carrying smaller molecules, but prone to undergo endocytosis when delivered with larger cargos [17]. In our observation, the penetration rate of SOD-Tat reached nearly 100% in RAW264.7 cells, but the penetrating rate of SOD1-EGFP-Tat was reduced to 30% in bone marrow cells. These variations may be explained by the differences in the molecular weights of cargos and the cell types subjected to treatment.

Oxidative stress can result from an accumulation of excess ROS. As one of the members of the antioxidative enzyme family, SOD1 has been widely investigated as a ROS-reducing reagent [13,14]. It was reported that the SOD1-Tat fusion protein could be internalized into multiple mammalian cells and that internalized SOD1-Tat increased the viability of cells pretreated with an intracellular ROS-generating reagent, indicating that, when fused with Tat, recombinant SOD1 proteins still protect cells from oxidative stress [18,19,20]. Consistently, in our study, the recombinant fusion protein SOD1-Tat showed antioxidant activity in both RAW264.7 cells and bone marrow LK cells.

Irradiation is a standard procedure during bone marrow transplant and it leads to a rapid increase in ROS in the mouse bone marrow microenvironment, which exacerbates bone marrow suppression and further decreases engraftment efficiency. Due to the weak hematopoietic state, the recipients are at a high risk of infection-related death, particularly in the first 2–8 weeks after BMT [1]. To increase the engraftment capability of donor cells and the self-renewal and reconstitution ability of the transplanted HSCs, ROS in the bone marrow niches should be kept at a low level. Strategies focusing on reducing ROS during BMT were explored and have shown promise in improving the engraftment capability of transplanted HSCs in recipient mice. Most of these studies reported that ROS-reducing interventions increased the engraftment efficacy at three weeks or later after BMT [4,5]. In our study, SOD1-Tat-pretreated donor BMCs exhibited significantly better engraftment capability at two weeks after BMT. In addition, treatment with SOD1-Tat may be safer than overexpression of ROS-detoxifying enzymes in donor cells. Moreover, donor BMCs pretreated with SOD1-Tat also exhibited a better engraftment ability than those pretreated with SOD1, indicating that SOD1-Tat prolongs the antioxidant effect compared with that of SOD1 alone, which could be explained by the fact that internalized SOD-Tat helps to maintain the antioxidant effect for a longer time. The uptake mechanism of SOD1-Tat in bone marrow cells and the long-term effect of this fusion protein on hematopoiesis remain to be investigated.

There are a few limitations associated with this study. First, the long-term effect of this fused CPP on donor cells should be further investigated in vitro and in vivo. Second, we only investigated the effect of SOD1-Tat on total bone marrow transplantation. This method may be applied to HSC transplantation (HSCT) since the HSCT model is also widely used in studying hematopoietic therapeutics. More precise manipulation of ROS might be achieved by selecting and treating donor HSCs with CPP-SOD1 for HSCT. Third, potential biases might be introduced by inter-heterogeneity with small group numbers, but these biases were offset to some extent by conducting competitive transplantation.

In summary, we demonstrated that the antioxidant protein SOD1 fused with the CPP Tat could increase the engraftment of total BMCs in irradiated mice. Thus, we provide some new insights for improving the efficiency of engraftment.

## 4. Materials and Methods

### 4.1. Mice

B6.SJL-Ptprca Pepcb/BoyJ mice carrying the CD45.1 allele and C57BL/6 mice carrying the CD45.2 allele were purchased from Jackson Laboratories. Mice carrying both CD45.1 and CD45.2 alleles were generated by crossing B6.SJL-Ptprca Pepcb/BoyJ mice and C57BL/6 mice. All animal experiments (License: SYXK2016-0167) were conducted according to the Animal Welfare Guidelines of the Southern Medical University of China and were supported by the Ethics Committee on the Use and Care of Animals, Southern Medical University.

### 4.2. Construction of Expression Vectors

A 6x-histidine tag was added to the pET14b vector (Novagen, Darmstadt, Germany, Cat. # 69660-3) by mutagenesis. The mouse SOD1 gene was amplified with a mouse cDNA library as a template and a pair of specific primers by polymerase chain reaction (PCR). The amplified SOD1 gene fragment was subsequently cloned into the pET14b-His vector with the Nde I and Kpn I enzyme sites for the construction of the His-tagged SOD1 prokaryotic expressing vector pET14b-His-SOD1. Similarly, prokaryotic expression vectors for His-tagged SOD1-Tat, SOD1-EGFP, and SOD1-EGFP-Tat were constructed. The coding sequence for Tat (49–57 amino acids) was inserted into the fusion protein expression vector by PCR. Detailed information on the primers used for PCR amplification is presented in Table 1. All the constructed expression vectors were verified by DNA sequencing (BGI, Beijing, China). The plasmids were amplified in DH5α Escherichia coli and purified with the HiPure Plasmid Miniprep Kit from Invitrogen (Carlsbad, CA, Cat. # K2100-11).

### 4.3. Preparation of Recombinant Fusion Proteins

The expression vectors were transformed into Rosetta 2(DE3) competent cells (Novagen, Darmstadt, Germany) for CPP fusion protein expression. The recombinant proteins were purified using a Ni2^+^-NTA Fast Start Kit from QIAGEN (Hilden, Germany, Cat. # 30600). A centrifugal filter unit from Millipore (Bedford, MA, USA, Cat. # UFC501008) was applied to remove the salt and to concentrate purified proteins. Protein quantitation was performed with a Pierce™ BCA protein assay kit from Thermo Fisher (Waltham, MA, USA, Cat. # 23227). Endotoxin was removed with endotoxin removal beads from Miltenyi Biotec (Germany, Cat. # 130-093-659). Endotoxin levels were determined by using a Chromogenic LAL Endotoxin Assay Kit from GenScript (Nanjing, China, Cat. # L00350).

### 4.4. Internalization and Imaging

Cultured RAW264.7 cells (1 × 10^4^) were incubated with 5 μΜ SOD1 or SOD1-Tat fusion protein at 37 °C for 1 h to observe CPP-mediated internalization. The cells were fixed with 4% paraformaldehyde (Sigma Aldrich, St Louis, MO, USA, Cat. # 158127) and permeabilized with 0.2% Triton X-100 (Sigma Aldrich, St Louis, MO, USA, Cat. # T8787). A specific antibody against SOD1 (Santa Cruz, CA, USA, Cat. #SC-101523) was incubated with the cells for 1 h, and then, the sample was washed with PBS three times. After incubation with an anti-mouse secondary antibody (Santa Cruz, CA, USA, Cat. #SC-2010) and DAPI (BD Biosciences, San Jose, CA, USA), the cells were examined using a fluorescence microscope (Carl Zeiss, Germany).

### 4.5. Detection of Hydrogen Peroxide

The ROS level was measured by flow cytometry with a DCFHDA probe of the CellROX^®^ green assay kit (ThermoFisher, Waltham, MA, USA, Cat. #C10492).

### 4.6. LK Cell Sorting

TBM cells from tibias and femurs were collected using 25-G needles and filtered with 40-µm cell strainers. Erythrocytes were lysed with ACK lysis buffer (ThermoFisher, Waltham, MA, USA, Cat. #A10492-01), and a white blood cell suspension was prepared. Lineage^-^c-Kit^+^ (LK) cells were sorted by flow cytometry as previously described [21].

### 4.7. Competitive Transplantation

Age-matched CD45.1 and CD45.2 C57BL/6 male mice were acclimated for 2 weeks after purchase from Jackson Laboratories. CD45.1^+^CD45.2^+^ mice were generated by cross mating CD45.1 and CD45.2 C57BL/6 mice. TBM cells were collected from the tibias and femurs of donors by spinning, followed by lysis of red blood cells using ACK lysis buffer (ThermoFisher, Waltham, MA, USA, Cat. #A1049201). Half a million BMCs from CD45.2 mice were treated with 5 μΜ recombinant SOD1-Tat, SOD1, or PBS for 1 h, and washed with PBS to remove residual recombinant proteins in the medium. Then, those CD45.2 BMC cells were mixed with an equal number of CD45.1 BMCs as competition to reconstitute the hematopoietic environment of a lethally irradiated recipient mouse (CD45.1^+^CD45.2^+^). The donor cells were transplanted into recipients via ophthalmic vein injection. Two weeks after transplantation, 100 µL of peripheral blood were collected from the recipients for complete blood count and hematopoietic chimerism analysis using a Hemavet hematology system (Drew Scientific, Inc., Miami Lakes, FL, USA). Cell sorting was performed by using a FACSCanto II flow cytometer (BD Biosciences, San Jose, CA, USA) [21,22].

### 4.8. FACS Analysis for Hematopoietic Chimerism

To evaluate engraftment and myeloid cell differentiation, peripheral blood cells were prepared and analyzed as previously described. Fluorescence-labeled CD45.2-APC (Cat. #109814), CD45.1-PE (Cat. #110708), Gr1-FITC (Cat. #108406), B220-APC/Cy7 (Cat. #103224), and CD3-PE/Cy7 (Cat. #1000320) were purchased from Biolegend, Inc. (San Diego, CA, USA), and DAPI was purchased from BD Biosciences (San Jose, CA, USA). These fluorescent antibodies were used to sort subsets of myeloid cells with a FACSCanto II flow cytometer (BD Biosciences, San Jose, CA, USA) [21,22].

### 4.9. Statistical Analysis

Analysis of statistical significance was performed using the unpaired 2-tailed Student’s t-test for comparison of the two groups or one-way ANOVA followed by LSD post hoc test for multiple comparisons with SPSS 23.0 (IBM, Armonk, NY, USA). Differences with *P* < 0.05 were considered statistically significant.

## Figures and Tables

**Table 1:** Detailed information of primers used for the construction of recombinant plasmids expressing SOD1, SOD1-Tat, SOD1-EGFP, or SOD1-EGFP-Tat.

Vector Name	Primer Name	Primer Sequence	Enzyme Site
**pET14b**	**SOD1**	Forward: 5′ ATAGATACATATGATGGCGATGAAAGCGGTGTGCG 3′	*Nde* I
		Reverse: 5′ TAGATGGTACCCTGCGCAATCCCAATCACTCCAC 3′	*Kpn I*
**pET14b**	**EGFP**	Forward: 5′ ATTAGGTACCGTGAGCAAGGGCGAGGAGCTGTTC 3′	*Kpn* I
		Reverse: 5′ ATATCTCGAGCTTGTACAGCTCGTCCATGCCGAGA 3′	*Xho* I
**pET14b**	**Tat**	Forward: 5′ GATCCTATGGCCGCAAGAAACGCCGTCAGCGTCGCCGTG 3′	*Bam*H I
		Reverse: 5′ GATCCACGGCGACGCTGACGGCGTTTCTTGCGGCCATAG 3′	*Bam*H I

## Data Availability

The data used to support the findings are available by contacting the corresponding author.
